# Characterization of the Leucocyte Immunoglobulin-like Receptor B4 (*Lilrb4*) Expression in Microglia

**DOI:** 10.3390/biology10121300

**Published:** 2021-12-09

**Authors:** Felix Kretzschmar, Robin Piecha, Jannik Jahn, Phani Sankar Potru, Björn Spittau

**Affiliations:** 1 Institute of Anatomy, Medicine Rostock, University of Rostock, 18055 Rostock, Germany; f.j.kretzschmar@googlemail.com (F.K.); Robin.Piecha@med.uni-rostock.de (R.P.); jannik.jahn@gmail.com (J.J.); phani.potru@uni-bielefeld.de (P.S.P.); 2 Anatomy and Cell Biology, Medical School OWL, Bielefeld University, 33615 Bielefeld, Germany

**Keywords:** Lilrb4, microglia, TGFβ1, LPS

## Abstract

**Simple Summary:**

In the present study, we provide a detailed characterization of *Lilrb4* expression in microglia and peripheral myeloid cells. Our data demonstrate that LILRB4 is a marker for microglia activation, as evidenced by upregulation after lipopolysaccharide treatment and inhibition of microglial TGFβ signaling. Moreover, we provide evidence that microglia express low levels of *Lilrb4* in vivo and high levels in vitro, and we clearly demonstrate that LILRB4 is also expressed by bone marrow-derived monocytes and, to a greater extent, by peritoneal macrophages, defining LILRB4 as a surface marker of myeloid cells and not as a microglia-specific marker.

**Abstract:**

As resident innate immune cells of the CNS, microglia play important essential roles during physiological and pathological situations. Recent reports have described the expression of *Lilrb4* in disease-associated and aged microglia. Here, we characterized the expression of *Lilrb4* in microglia in vitro and in vivo in comparison with bone marrow-derived monocytes and peritoneal macrophages in mice. Using BV2 cells, primary microglia cultures as well as ex vivo isolated microglia and myeloid cells in combination with qPCR and flow cytometry, we were able to provide a comprehensive characterization of *Lilrb4* expression in distinct mouse myeloid cells. Whereas microglia in vivo display low expression of *Lilrb4*, primary microglia cultures present high levels of surface LILRB4. Among the analyzed peripheral myeloid cells, peritoneal macrophages showed the highest expression levels of *Lilrb4*. Moreover, LPS treatment and inhibition of microglial TGFβ signaling resulted in significant increases of LILRB4 cell surface levels. Taken together, our data indicate that LILRB4 is a reliable surface marker for activated microglia and further demonstrate that microglial TGFβ signaling is involved in the regulation of *Lilrb4* expression during LPS-induced microglia activation.

## 1. Introduction

Microglia are specialized resident innate immune cells which mediate immune surveillance of the central nervous system (CNS) and play important roles under physiological and pathological conditions [1,2]. During early embryonic development, PU.1 and IRF8 are necessary for primitive macrophages to arise from the yolk sac [3,4]. Prior to birth, these microglia precursor cells actively migrate toward the developing CNS in dependence of the interleukin-34 (IL-34) and colony-stimulating factor 1 receptor (CSF1R) ligand-receptor axis [5,6]. Within the first postnatal weeks, microglia mature and start to establish a unique and cell-specific gene expression signature that clearly distinguishes these resident CNS immune cells from other macrophage populations and is characterized by the increased expression of genes such as *transmembrane protein 119* (*Tmem119*), *purinergic receptor P2Y12* (*P2ry12*), *olfactomedin-like 3* (*Olfml3*), *sal-like 1* (*Sall1*), *G-protein receptor 34* (*Gpr34*), *hexosaminidase beta* (*Hexb*), and *Fc receptor-like S* (*Fcrls*) [7,8,9,10]. Interestingly, *Tmem119* and *Olfml3* have been described to be direct TGFβ1-Smad2 target genes, and recent studies have confirmed that the microglia maturation process is dependent on neural TGFβ1, microglial TGFβ signaling, and proper extracellular TGFβ1 processing and binding [9,11,12,13]. TGFβ1 has been proven to be a central factor for microglia homeostasis and maintenance, regulating immune reactions and activation states of microglia in vitro and in vivo [14,15]. Microglia are constantly controlling their local microenvironment, reacting to disturbances triggered by endogenous and/or exogenous factors [16]. Damage-associated molecular patterns (DAMPs) and pathogen-associated molecular patterns (PAMPs) have been classified and are sensed by microglial surface receptors, including Toll-like receptors (TLRs) or NOD-like receptors (NLRs), resulting in microglia activation [17]. Initially, microglia activation was compared with macrophage M1 and M2 polarization, and several in vitro studies supported this classification of microglia activation [18,19,20,21]. However, sophisticated in vivo studies using (single-cell) RNA sequencing have resulted in the description of a highly conserved transcriptional profile under neurodegenerative conditions, including the upregulation of *ApoE*, *Axl*, *Clec7a*, *Cst7*, *Cybb*, *Ctsd*, *Il1b*, *Itgax*, *Lgals*, *Lilrb4*, *Lpl*, *Nos2*, *Spp1*, *Trem2* as well as *Tyrobp* [22,23]. Based on these data, microglia are present as homeostatic microglia under basal conditions and adopt an activation profile under pathological conditions that characterizes them as disease-associated microglia (DAM). Depending on the pathology and the severity, these DAMs can further shift toward neurodegenerative microglia (MGnDs) [24]. It is noteworthy that several studies have demonstrated that DAMs and aged microglia show an overlapping expression profile which includes upregulation of the *leucocyte immunoglobulin-like receptor B4* (*Lilrb4*) [25,26,27].

LILRB4, also referred to as CD85k, ILT3, or GP49B, belongs to the family of leucocyte immunoglobulin (Ig)-like receptors (LILRs), which are able to associate with membrane-anchored adaptors to induce signaling via cytoplasmic immunoreceptor tyrosine-based inhibitory motifs (ITIMs). Thus, expression of *Lilrb4* is considered to mediate inhibitory and immunoregulatory functions in the distinct immune cell type [28]. Expression of Lilrb4 has been demonstrated in a plethora of peripheral immune cells including B cells, T cells, dendritic cells, mast cells, macrophages, and monocytes [28]. Although the ligands for human LILRB4 remain unknown, murine LILRB4 has been described to interact with integrin avb3 [29,30]. However, the functions of LILRB4 in the context of immunologic responses is not well understood. Studies with *Lilrb4*-deficient mice have demonstrated exacerbated responses to bacterial lipopolysaccharide (LPS) in a model for acute lung injury and increased cytokine and chemokine productions in LPS-induced synovitis [31,32].

In the present study, we provide a comprehensive characterization of LILRB4 expression in mouse microglia in vitro and in vivo in comparison with peritoneal macrophages and bone marrow-derived monocytes. We could clearly demonstrate the microglia show a distinct but weaker LIRLB4 surface expression compared with peripheral immune cells. Moreover, we show that LPS induces upregulation of *Lilrb4* in microglia and that inhibition of TGFβ signaling also results in increased expression of LILRB4. Together, our data indicate increased expression of *Lilrb4* as a hallmark of microglia activation and define LILRB4 as a common immunologic surface receptor of microglia and peripheral monocytes/macrophages.

## 2. Materials and Methods

### 2.1. Animals

NMRI mice used for establishment of primary microglia cultures as well as for the isolation of peritoneal macrophages and bone marrow-derived monocytes at indicated postnatal stages were purchased from Janvier (Le Genest-Saint-Isle, France). All mice were kept at 22 ± 2 °C under a 12 h light/dark cycle with ad libitum access to chow and water. Animal experiments were conducted in accordance with the German Federal Animal Welfare Law and local ethical guidelines of the University of Rostock. Experiments involving mice have been approved by the animal experimentation committee of the University of Rostock and the Landesamt für Landwirtschaft, Lebensmittelsicherheit und Fischerei Mecklenburg-Vorpommern (7221.3-1-064/18).

### 2.2. Reagents

Cultures of primary microglia and BV2 cells were treated with the following factors and reagents: TGFβ1 (100-21C, Peprotech, Hamburg, Germany) 5 ng/mL, LPS (L8274, Sigma-Aldrich, Schnelldorf, Germany) 1 µg/mL, TGFβ receptor type I inhibitor (TβRI, 616454, Calbiochem, Merck, Darmstadt, Germany) 500 nM.

### 2.3. Microglia Cultures

Cultures of primary microglia were generated as described previously [14]. Brains from P0/P1 NMRI mice were washed with Hank’s balanced salt solution (HBSS, 240201117, Thermo Fisher Scientific, Bremen, Germany) and meninges and vessels were removed. Dissected brains were collected in ice-cold HBSS and further digested using 1× Trypsin-EDTA (25300054, Invitrogen, Darmstadt, Germany) for 10 min at 37 °C. An equal amount of ice-cold fetal calf serum (FCS) and DNase (M0303S, New England BioLabs, Frankfurt/Main, Germany) at a final concentration of 0.5 mg/mL were added before dissociation of the brains with Pasteur pipettes. Dissociated cells were centrifuged, collected and resuspended in DMEM/F12 medium containing 10% FCS and 1% penicillin/streptomycin (P06-07050, PAN Biotech, Aidenbach, Germany). Finally, cells were transferred into poly-L-lysine-coated (P2636-25MG, Sigma-Aldrich, Schnelldorf, Germany) tissue culture flasks with a density of 2–3 brains per 75 cm^2^ flask or 1 brain per 25 cm^2^ flask.

### 2.4. BV2 Cell Culture

The mouse microglia cell line BV2 was cultured in DMEM/F12 (11320033, Gibco, Darmstadt, Germany) supplemented with 10% heat-inactivated FCS and 1% penicillin/streptomycin (Sigma-Aldrich, Schnelldorf, Germany). Cells were incubated at 37 °C in a 5% CO_2_ and 95% humidified atmosphere. Prior to serum-free treatment with TGFβ1 and/or LPS, BV2 cells were rinsed with PBS and kept under serum-free conditions for at least 2 h prior to further treatment.

### 2.5. Ex Vivo Microglia Isolation

Ex vivo isolation of microglia was performed on 7 day (P7) and 30 day (P30) old male and female NMRI mice, as recently described [33]. Mice were killed by cervical dislocation, and brains were immediately dissected, washed with ice-cold PBS and collected in cold buffer (1x HBSS, 1% BSA, 1 mM EDTA). Brains were homogenized using a glass homogenizer and filtered through a 70 µm cell strainer (Falcon, Fisher Scientific, Bremen, Germany). After centrifugation (12 min at 300× *g*, 4 °C), the cell pellet was resuspended in 5 mL 37% Percoll (P1644, Sigma-Aldrich) in PBS, underlaid with 4 mL 70% Percoll solution and overlaid with 4 mL 30% Percoll in a 15 mL tube. Gradients were centrifuged for 30 min at 600× *g* and 4 °C without acceleration and deceleration. Finally, the microglia cell layer was collected from the 70% and 37% Percoll interface and transferred to FACS buffer (PBS, 1% FCS).

### 2.6. Isolation of Peritoneal Macrophages (PMs) and Bone Marrow-Derived Monocytes (BMMCs)

Peritoneal macrophages (PM) were isolated by the modified protocol published by Koerten et al. [34]. A total of 3–4 mL PBS was injected into the peritoneal cavity of NMRI mice, and the abdomen was tapped several times to release macrophages from peritoneal surfaces. Finally, PBS containing PMs was collected in a 15 mL tube and an equal volume of FACS buffer was added. Bone marrow-derived monocytes (BMMCs) were collected using the protocol reported by Wagner and colleagues [35]. Briefly, the femurs of NMRI mice were dissected, disinfected with 96% ethanol and perfused with 4 mL PBS. The suspension was collected in a 15 mL tube and an equal volume of FACS buffer was added.

### 2.7. Flow Cytometry

Microglia, peritoneal macrophages (PMs) and bone marrow-derived monocytes (BMMCs) were incubated with primary antibodies directed against F4/80 (5 µL, MCA497A488, AbD Serotech, dilution 1:40) and LILRB4 (144906, Biolegend, San Diego, CA, USA, dilution 1:40) at 4 °C for 15 min. Fc receptor blocking was performed for all samples using TrueStain fcX (101319, BioLegend, dilution 1:20) to avoid unspecific antibody binding. Finally, cells were rinsed and analyzed using a CytoFlex cytometer (Beckman Coulter, Krefeld, Germany) and the CytExpert software (version 2.4, Beckman Coulter, Krefeld, Germany).

### 2.8. RNA Isolation, Reverse Transcription and Quantitative RT-PCR

After treatment, total RNA was isolated from BV2 cells and primary microglia using TRIzol (15596026, Invitrogen, Darmstadt, Germany) according to the manufacturer’s instructions. RNA concentrations were determined using Photometer (Eppendorf BioPhotometer D30). Reverse transcription was performed using the ProtoScript II First Strand cDNA Synthesis Kit (M0368L, New England BioLabs, Frankfurt/Main, Germany) according to the manufacturer´s instructions. Quantitative RT-PCR (qPCR) was performed using the CFX Connect System (Bio-Rad, München, Germany) in combination with the Luna Universal qPCR Master Mix (M3003L, New England BioLabs). All qPCR reactions were performed in duplicates and results were analyzed using the CFX Connect System software and the comparative CT method. All data are presented as 2^−∆∆CT^ for the gene of interest (*Lilrb4*) normalized to the housekeeping gene *Gapdh* and presented as fold change relative to the control groups. The following primers have been used: Lilrb4*for* 5′-ATGGGCACAAAAAGAAGGCTAA-3′, Lilrb4*rev* 5′-GGCATAGGTTACATCCTGGGTC-3′(NM_013532.3), Gapdh*for* 5′;-AGGTCGGTGTGAACGGATTTG-3′, Gapdhrev 5′-TGTAGACCATGTAGTTGAGGTCA-3′(NM_008084).

### 2.9. Statistics

All presented data are given as means ± SEM. Multiple group analysis for samples with variance homogeneity was performed using one-way ANOVA followed by Tukey´s multiple comparison test. Samples with lack of variance homogeneity were analyzed using the nonparametric Kruskal–Wallis test followed by Dunn´s multiple comparison test. *p-*values < 0.05 were considered to be statistically significant. Statistical analyses were conducted using GraphPad Prism 8 (GraphPad Software Inc., San Diego, CA, USA).

## 3. Results

### 3.1. Expression of Lilrb4 in Primary Microglia In Vitro

**Figure 1:**
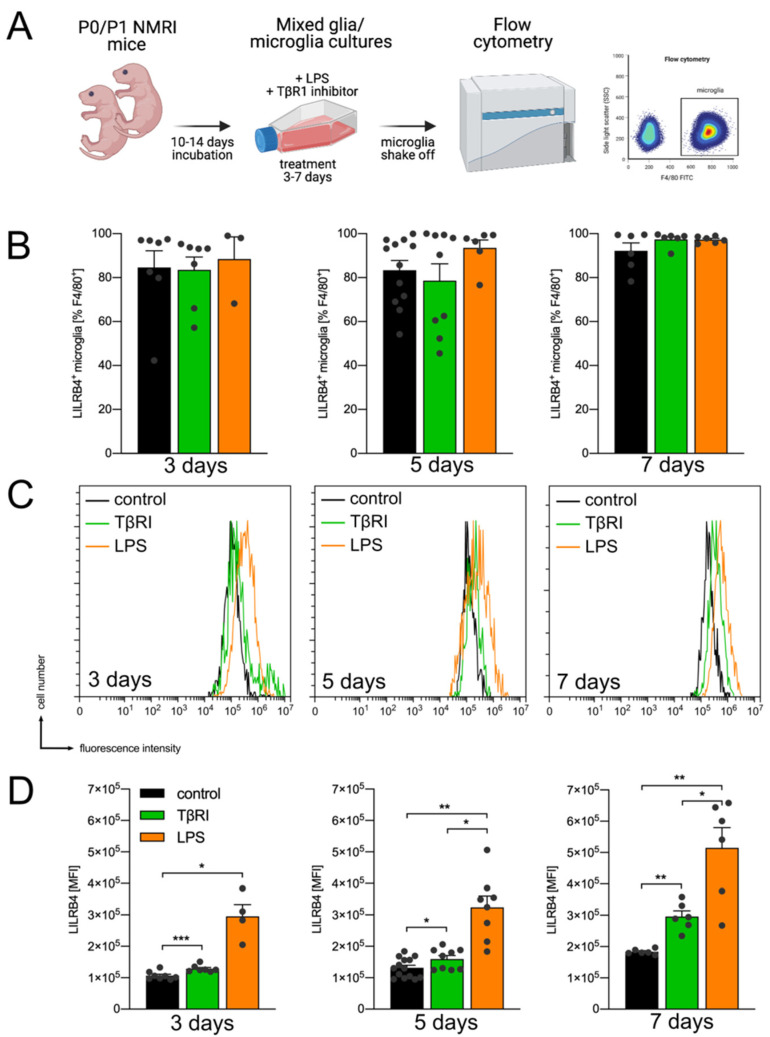
Expression of *Lilrb4* in primary microglia. Schematic of the experimental design to analyze LILRB4 expression in primary microglia. Created with BioRender.com (**A**). Percentages of LILRB4^+^ microglia (out of F4/80^+^ cells) after 3, 5, and 7 days (**B**). Representative flow cytometry histograms of control cells and after treatment of microglia with TβRI and LPS (**C**). Quantifications and statistical analyzes of mean fluorescence intensities (MFI) of LILRB4 after indicated treatments and time points (**D**). All data are given as means ± SEM for at least three independent experiments. *p*-values derived from nonparametric Kruskal–Wallis tests followed by Dunn’s multiple comparison tests are * *p* < 0.05, ** *p* < 0.01, and *** *p* < 0.001.

In order to address the question whether *Lilrb4* is expressed in microglia, primary mouse microglia were used. As depicted in Figure 1A, mixed glia/microglia cultures were treated with a TGFβ receptor inhibitor (TβRI 500 nM) or lipopolysaccharide (LPS, 1 µg/mL) for 3, 5 and 7 days. Microglial surface expression of LILRB4 was analyzed after microglia shake off from mixed cultures using flow cytometry. Figure 1B shows that most F4/80^+^ microglia were also positive for LILRB4 under basal conditions (84.59% ± 7.585%) and further treatment with TβRI (83.56% ± 5.855%) or LPS (88.45% ± 10.11%) did not result in significant changes in LILRB4^+^ microglia. However, a strong tendency toward increased numbers of F4/80^+^/LILRB4^+^ microglia was detected after treatment with TβRI (97.41% ± 1.333%) and LPS (97.28% ± 0.61%) for 7 days. To detect differences in total LILRB4 surface levels, the mean fluorescence intensities (MFI) were analyzed for the indicated time points and treatments (Figure 1C,D). Flow cytometry histograms indicate that treatment with TβRI and LPS resulted in increased LILRB4 fluorescence intensity in microglia (Figure 1C). Quantifications revealed that treatment with TβRI and LPS resulted in significant increases of LILRB4 fluorescence intensities after 3 days (control = 106,587 ± 4579, TβRI = 128,808 ± 4100, LPS = 295,697 ± 36,951), 5 days (control = 131,503 ± 8375, TβRI = 159,147 ± 10,956, LPS = 324,011 ± 35,995), and 7 days (control = 183,403 ± 3417, TβRI = 295,910 ± 17,962, LPS = 514,716 ± 64,637). Of note, LPS treatments always provoked a stronger LILRB4 fluorescence intensity as compared to inhibition of TGFβ signaling, reaching statistical significances after 5 days (*p* = 0.0278) and 7 days (*p* = 0.0168, Figure 1D). Together, these data demonstrate that virtually all primary microglia express *Lilrb4* and show LILRB4 surface localization. Moreover, microglia activation induced by LPS or pharmacological inhibition of microglial TGFβ signaling resulted in time-dependent increases in LILRB4 on microglia surfaces, further indicating upregulation of *Lilrb4* during microglia activation processes.

### 3.2. TGFβ1 Inhibits LPS-Mediated Transcriptional Upregulation of Lilrb4

**Figure 2:**
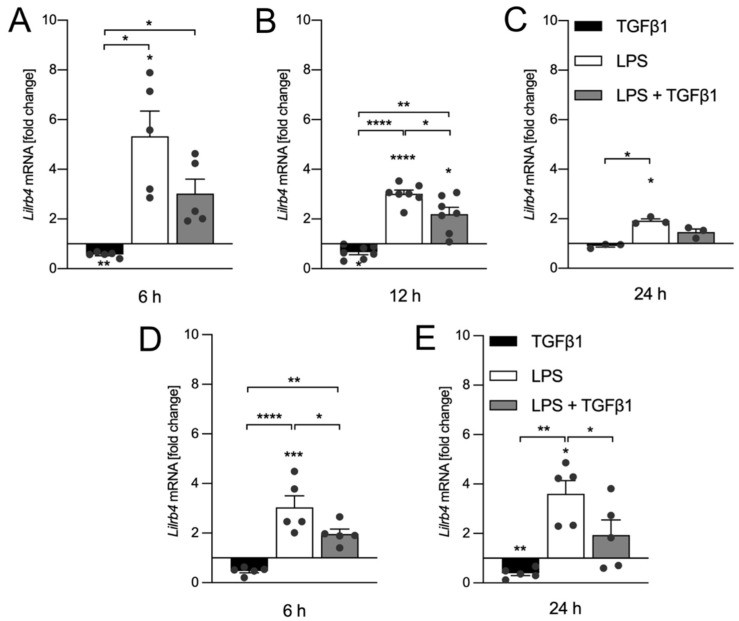
TGFβ1 inhibits LPS-induced upregulation of Lilrb4 in BV2 cells and primary microglia. Expression of Lilrb4 after treatment of BV2 cells with TGFβ1 (5 ng/mL), LPS (1 µg/mL) or the combination of both factors (LPS + TGFβ1) for 6 h (**A**), 12 h (**B**), and 24 h (**C**). Expression of Lilrb4 after treatment of primary microglia with TGFβ1 (5 ng/mL), LPS (1 µg/mL) or the combination of both factors (LPS + TGFβ1) for 6 h (**D**) and 24 h (**E**). Data are given as means ± SEM for at least three independent experiments. *p*-values derived from one-way ANOVA followed by Tukey’s multiple comparison tests are * *p* < 0.05, ** *p* < 0.01, *** *p* < 0.001, and **** *p* < 0.0001.

After the observation that LPS treatment as well as inhibition of TGFβ signaling increased LILRB4 surface expression, we used the microglia cell line BV2 as well as primary microglia cultures to address whether treatment with recombinant TGFβ1 is able to interfere with the LPS-induced upregulation of *Lilrb4*. Therefore, BV2 cells were treated either with TGFβ1 (5 ng/mL), LPS (1 µg/mL) or the combination of both factors for 6 h, 12 h, and 24 h. As shown in Figure 2A, TGFβ1 treatment significantly downregulated (*p* = 0.0025) the expression of *Lilrb4* but did not significantly inhibit the LPS-induced upregulation of *Lilrb4* in BV2 cells after 6 h. Similar results were obtained after treatment for 12 h. TGFβ1 treatment significantly downregulated (*p* = 0.0497) the expression of *Lilrb4* and significantly blocked (*p* = 0.0244) the LPS-induced upregulation (Figure 2B). After 24 h, LPS treatment resulted in a significant (*p* = 0.0198) but less pronounced upregulation of *Lilrb4*, and TGFβ1 treatment seemed to interfere with the LPS effects without reaching statistical significance. However, in this case, the conclusion could be affected by the relatively small number of samples per group. In order to validate these results, primary microglia cultures were further treated either with TGFβ1 (5 ng/mL), LPS (1 µg/mL) or the combination of both factors for 6 h and 24 h. Figure 2D shows that TGFβ1 significantly inhibited (*p* = 0.0305) the LPS-induced upregulation (*p* = 0.0002) of *Lilrb4* in primary microglia after 6 h. After treatment for 24 h, TGFβ1 alone significantly downregulated (*p* = 0.0091) *Lilrb4* expression and further significantly abrogated (*p* = 0.0488) the increase of *Lilrb4* expression triggered by LPS. In summary, TGFβ1 treatment alone results in rapid downregulation of *Lilrb4* and further blocks the LPS-mediated transcriptional upregulation of *Lilrb4* in BV2 cells.

### 3.3. Lilrb4 Is Highly Expressed in Peripheral Macrophages

**Figure 3:**
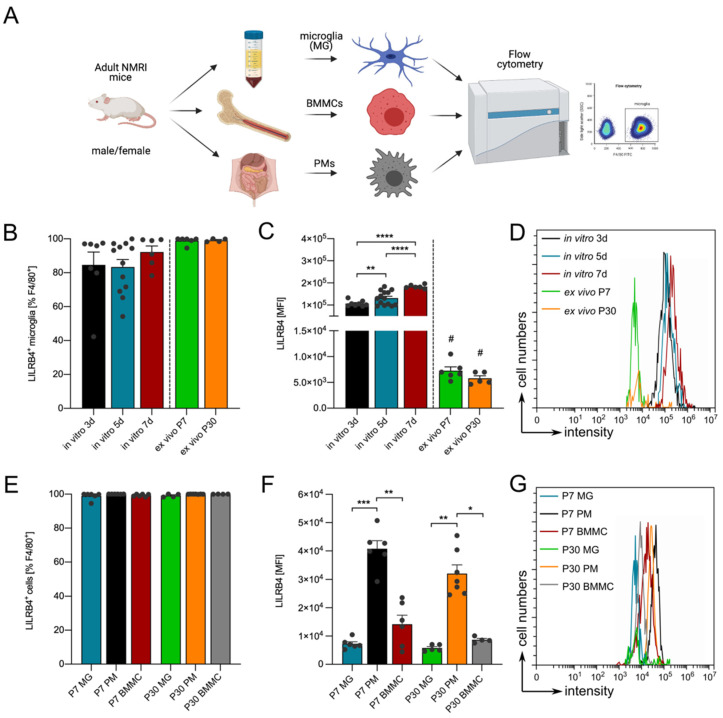
LILRB4 is highly expressed in peritoneal macrophages. Schematic of the experimental design to analyze LILRB4 expression in microglia, bone marrow-derived monocytes (BMMCs), and peritoneal macrophages (PMs). Created with BioRender.com (**A**). Percentages of LILRB4^+^ microglia (out of F4/80^+^ cells) after 3, 5, and 7 days in vitro as well as 7 days and 30 days ex vivo (**B**). Quantifications and statistical analyses of mean fluorescence intensities (MFI) of LILRB4 at indicated time points (**C**). Representative flow cytometry histograms (**D**). Comparison of percentages of LILRB4^+^ microglia, PMs, and BMMCs (**E**). Quantifications and statistical analyses of MFIs of LILRB4^+^ microglia, PMs, and BMMCs (**F**). Representative flow cytometry histograms (**G**). All data are given as means ± SEM for at least three independent experiments. *p*-values derived from one-way ANOVA followed by Tukey’s multiple comparison tests (**E**,**F**) or nonparametric Kruskal–Wallis tests followed by Dunn´s multiple comparisons tests (**B**,**C**) are # *p* < 0.05, * *p* < 0.05, ** *p* < 0.01, *** *p* < 0.0001, and **** *p* < 0.00001.

To address the question of whether the expression of *Lilrb4* is restricted to microglia, NMRI mice (7 days old (P7) and 30 days old (P30)) were used for further experiments, and primary microglia, bone marrow-derived monocytes (BMMCs), and peritoneal macrophages (PMs) were acutely isolated as described in the material and methods section; additionally, surface expression of LILRB4 was analyzed using flow cytometry (Figure 3A). A shown in Figure 3B, virtually all ex vivo isolated microglia from P7 (98.86% ± 0.879%) and P30 (99.16% ± 0.46%) mice were positive for LILRB4 and, thus, were similar to in vitro cultured primary microglia. However, analysis of the MFIs revealed significantly lower expression levels of *Lilrb4* in ex vivo isolated microglia (P7 = 7262 ± 731.1, P30 = 5777 ± 509) compared to cultured primary cells after 3 days (106,587 ± 4579), 5 days (131,503 ± 8375), and 7 days (183,403 ± 3417) in vitro (Figure 3C,D). Moreover, the expression of LILRB4 in ex vivo microglia seemed to decrease as microglia mature, whereas primary microglia in vitro increased LILRB4 surface expression levels after longer incubation periods (Figure 3C). Next, we compared the percentages of LILRB4^+^ microglia in P7 (98.86% ± 0.879%) and P30 (99.16% ± 0.46%) with aged-matched BMMCs and PMs. Similar to microglia, all BMMCs (99.98% ± 0.02%) as well as PMs (99.98% ± 0.015%) are LILRB4-positive (Figure 3E).

**Figure 4:**
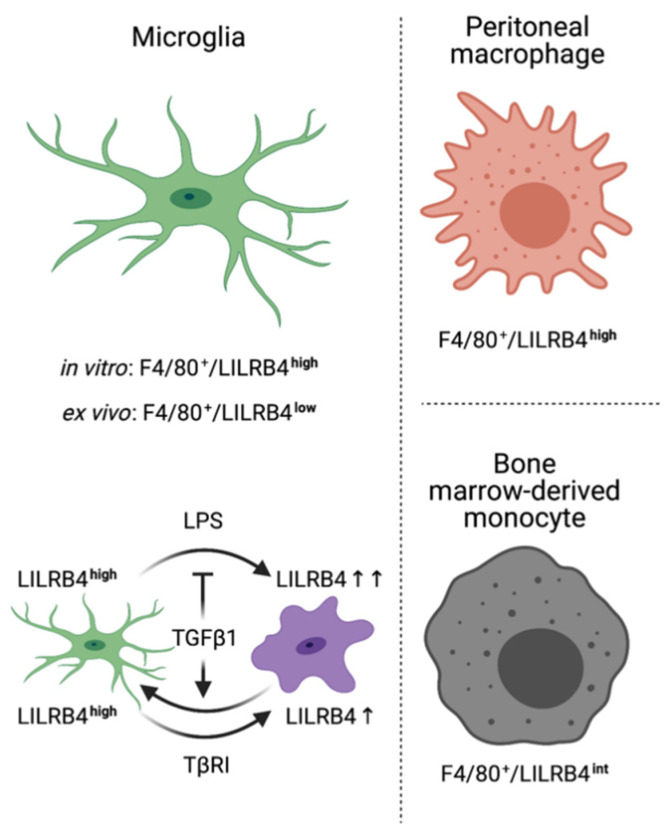
Summary scheme depicting expression patterns of LILRB4 in microglia, peritoneal macrophages and bone marrow-derived monocytes. Created with BioRender.com.

Comparison of MFIs clearly demonstrated that peritoneal macrophages (PMs) display the highest expression levels and surface localizations of LILRB4 among these different cell types at P7 (40,751 ± 2855) as well as P30 (32,073 ± 3018, Figure 3F,G). Taken together, these data indicate that expression of *Lilrb4* is not restricted to microglia, and that BMMCs and PMs also show *Lilrb4* expression. Of note, the highest expression of *Lilrb4* was detected in peritoneal macrophages, and the lowest expression levels were obtained in microglia. A summary scheme for LILRB4 surface expression and regulation by LPS and TGFβ signaling is given in Figure 4.

## 4. Discussion

In the present study, we have provided a detailed characterization of *Lilrb4* expression in mouse microglia, peritoneal macrophages and bone marrow-derived monocytes. Using flow cytometry, we were able to demonstrate that all of the abovementioned immune cells display LILRB4 on their cell surfaces. However, expression levels vary among distinct subsets of cells. Whereas primary microglia and bone marrow-derived monocytes show low surface expression of LILRB4, peritoneal macrophages showed the highest levels of surface LILRB4. A detailed comparison of primary microglia from in vitro cultures and acutely ex vivo isolated microglia from postnatal mice revealed that the expression of LILRB4 was detected in virtually all microglia, at least at low levels. The most striking result obtained here was that microglia from in vitro cultures showed the highest expression of LILRB4 amongst all cell types analyzed throughout this study. Interestingly, expression levels significantly increased depending on the duration of microglia culturing. Moreover, the surface levels of microglial LILRB4 further increased in vitro after treatment with LPS or inhibition of microglial TGFβ signaling under serum-containing culture conditions. Using the microglia cell line BV2, we could demonstrate that treatment with LPS induces rapid transcriptional upregulation of *Lilrb4* which was inhibited by additional treatment with recombinant TGFβ1. Overall, our data indicate that LILRB4 is a reliable marker for microglia activation, but not a microglia-specific surface molecule, and further suggest that TGFβ signaling is involved in inhibition of *Lilrb4* upregulation during the microglia activation process.

The first reports showing upregulation of *Lilrb4* in microglia analyzed the gene expression signature of disease-associated microglia (DAM) in vivo [26,27] and, thus, this surface receptor is part of the DAM molecular signature [24]. Moreover, high expression of *Lilrb4* has further been reported in aged mice, indicating that aged microglia and DAMs show at least in part a similar expression pattern [23,25]. Interestingly, conditional knockout of the essential TGFβ signaling receptor *Tgfbr2* in microglia resulted in strong upregulation of *Lilrb4* in microglia with impaired TGFβ signaling [15]. Based on these results, we analyzed the effect of TGFβ signaling on expression of *Lilrb4* in primary microglia in vitro. The results presented in the present study clearly reveal that TGFβ1 alone transcriptionally downregulates *Lilrb4* and further blocks LPS-induced upregulation of *Lilrb4* in BV2 cells. Pharmacologic inhibition of TGFβ signaling over several days resulted in increasing LILRB4 surface levels in primary microglia. Given that LILRB4 is a microglia activation marker, our results further underline the essential role of TGFβ1 as an anti-inflammatory and immune-modulatory factor for microglia in vitro and in vivo [14,20,36,37]. It remains unclear whether *Lilrb4* is a direct target gene for microglial TGFβ signaling or whether the TGFβ1-induced downregulation is an indirect effect of TGFβ interfering with inflammatory signaling pathways.

It is difficult to speculate about the possible functions of LILRB4 in microglia. Recent studies addressing LILRB4 functions using *Lilrb4* knockout mice have exclusively focused on peripheral immune cells in distinct disease models. Lack of *Lilrb4* resulted in exaggerated LPS-induced intravascular aggregation of neutrophils which subsequently caused cutaneous microangiopathy in a model of proliferative synovitis. Moreover, total amounts of IL-1β, macrophage inflammatory protein 1α (MIP-1α), and MIP-2 were significantly higher in joint extracts from *Lilrb4*-deficient mice [31]. A study using atherosclerotic lesions from human coronary arteries described upregulated expression of *LILRB4* in macrophages. In aortic roots from mice with *Lilrb4* deficiency, significantly accelerated development of atherosclerotic lesions and increased instability of plaques associated with increased infiltration of lipids and decreased collagen components and smooth muscle cells were observed. Interestingly, the development of atherosclerosis was promoted by *Lilrb4*-deficient bone marrow-derived monocytes, which triggered pro-inflammatory effects by increased activation of NF-κB signaling due to decreased Shp1 phosphorylation [38]. Similar results were obtained in a model for acute lung injury (ALI) induced by LPS. Loss of LILRB4 exacerbated ALI and enhanced lung inflammation by inflammatory bone marrow-derived monocytes/macrophages (BMDMs) displaying increased activation of NF-κB signaling [32]. In microglia, activation of NF-κB signaling has been extensively described as a hallmark of reactive microglia and disease-associated microglia (DAMs) [39]. Interestingly, upregulation of the protein tyrosine phosphatase Shp1 has been reported in activated microglia, and Shp1-mutant mice show enhanced microglia and astroglia activation [40]. Furthermore, LPS-induced activation of microglia with reduced Shp1 activity resulted in the increased release of nitric oxide (NO), tumor necrosis factor α (TNFα) and IL-1β [41]. These data indicate that upregulation of *Lilrb4* might be involved in regulating the extent of microglial reactivity and the associated cytokine release after LPS treatment. Recent data further suggest that LILRB4 suppresses Fc receptor-dependent monocyte functions via its ITIMs [30], and co-ligation of LILRB4 with FcγRI was associated with suppression of Fc receptor-dependent uptake of antibody-opsonized bacterial particles [42]. This could put LILRB4 functions in microglia in context with engulfment of pathogens and/or cellular debris and myelin and might explain the observed *Lilrb4* upregulation in aged cortical microglia [25]. Genome-wide association studies of late-onset Alzheimer’s disease risk predicted *OAS1*, *LAPTM5*, *ITGAM/CD11b* and *LILRB4* as four new risk genes for this neurodegenerative disease. In particular, *LILRB4* showed increased transcripts in the presence of amyloid plaques similar to the increase of the average microglial transcripts and the increase in microglia numbers [43]. These data suggest that amyloid plaques can trigger microglial *LILRB4* upregulation and—based on the potential role of LILRB4 during Fc receptor-mediated phagocytosis—might be involved in the engulfment of amyloid.

However, the molecular mechanisms of microglial LILRB4 function, the interaction partners as well as the involved intracellular signaling components remains unclear and need to be elucidated in future studies employing *Lilrb4*-deficient mice. Taking advantage of newly developed sophisticated techniques, microglia phenotypes in conditional knockout mice using microglia-specific Cre lines such as *Tmem119-Cre* or *Hexb-CreERT2* [44,45] to silence *Lilrb4* will further elucidate the role of LILRB4 in microglia under physiological and pathological conditions.

Taken together, the present study provides a detailed characterization of *Lilrb4* expression in microglia. Our data demonstrate that LILRB4 is a marker for microglia activation, as evidenced after treatment with LPS and inhibition of microglial TGFβ signaling. Further, we provide evidence that microglia express low levels of *Lilrb4* in vivo and high levels in vitro, supporting the notion that primary microglia cultures in vitro are pre-activated. Moreover, we could clearly demonstrate that LILRB4 is also expressed by bone marrow-derived monocytes and to a greater extent by peritoneal macrophages, defining LILRB4 as a surface marker of myeloid cells and not as a microglia-specific marker.

## Data Availability

The data that support the findings of this study are available from the corresponding author upon reasonable request.

## References

[1] Prinz M., Jung S., Priller J. (2019). Microglia Biology: One Century of Evolving Concepts. Cell.

[2] Prinz M., Priller J. (2014). Microglia and brain macrophages in the molecular age: From origin to neuropsychiatric disease. Nat. Rev. Neurosci..

[3] Kierdorf K., Erny D., Goldmann T., Sander V., Schulz C., Perdiguero E.G., Wieghofer P., Heinrich A., Riemke P., Hölscher C. (2013). Microglia emerge from erythromyeloid precursors via Pu.1- and Irf8-dependent pathways. Nat. Neurosci..

[4] Goldmann T., Wieghofer P., Jordão M.J.C., Prutek F., Hagemeyer N., Frenzel K., Amann L., Staszewski O., Kierdorf K., Krueger M. (2016). Origin, fate and dynamics of macrophages at central nervous system interfaces. Nat. Immunol..

[5] Ginhoux F., Greter M., Leboeuf M., Nandi S., See P., Gokhan S., Mehler M.F., Conway S.J., Ng L.G., Stanley E.R. (2010). Fate mapping analysis reveals that adult microglia derive from primitive macrophages. Science.

[6] Greter M., Lelios I., Pelczar P., Hoeffel G., Price J., Leboeuf M., Kündig T.M., Frei K., Ginhoux F., Merad M. (2012). Stroma-derived interleukin-34 controls the development and maintenance of langerhans cells and the maintenance of microglia. Immunity.

[7] Bennett M.L., Bennett F.C., Liddelow S.A., Ajami B., Zamanian J.L., Fernhoff N.B., Mulinyawe S.B., Bohlen C.J., Adil A., Tucker A. (2016). New tools for studying microglia in the mouse and human CNS. Proc. Natl. Acad. Sci. USA.

[8] Attaai A., Neidert N., von Ehr A., Potru P.S., Zöller T., Spittau B. (2018). Postnatal maturation of microglia is associated with alternative activation and activated TGFβ signaling. Glia.

[9] Butovsky O., Jedrychowski M.P., Moore C.S., Cialic R., Lanser A.J., Gabriely G., Koeglsperger T., Dake B., Wu P.M., Doykan C.E. (2014). Identification of a unique TGF-β-dependent molecular and functional signature in microglia. Nat. Neurosci..

[10] Hickman S.E., Kingery N.D., Ohsumi T.K., Borowsky M.L., Wang L., Means T.K., El Khoury J. (2013). The microglial sensome revealed by direct RNA sequencing. Nat. Neurosci..

[11] Arnold T.D., Lizama C.O., Cautivo K.M., Santander N., Lin L., Qiu H., Huang E.J., Liu C., Mukouyama Y.-S., Reichardt L.F. (2019). Impaired αVβ8 and TGFβ signaling lead to microglial dysmaturation and neuromotor dysfunction. J. Exp. Med..

[12] Qin Y., Garrison B.S., Ma W., Wang R., Jiang A., Li J., Mistry M., Bronson R.T., Santoro D., Franco C. (2018). A Milieu Molecule for TGF-β Required for Microglia Function in the Nervous System. Cell.

[13] Spittau B., Dokalis N., Prinz M. (2020). The Role of TGFβ Signaling in Microglia Maturation and Activation. Trends Immunol..

[14] Spittau B., Wullkopf L., Zhou X., Rilka J., Pfeifer D., Krieglstein K. (2013). Endogenous transforming growth factor-beta promotes quiescence of primary microglia in vitro. Glia.

[15] Zöller T., Schneider A., Kleimeyer C., Masuda T., Potru P.S., Pfeifer D., Blank T., Prinz M., Spittau B. (2018). Silencing of TGFβ signalling in microglia results in impaired homeostasis. Nat. Commun..

[16] Nimmerjahn A., Kirchhoff F., Helmchen F. (2005). Resting microglial cells are highly dynamic surveillants of brain parenchyma in vivo. Science.

[17] Kigerl K.A., de Rivero Vaccari J.P., Dietrich W.D., Popovich P.G., Keane R.W. (2014). Pattern recognition receptors and central nervous system repair. Exp. Neurol..

[18] Colton C.A. (2009). Heterogeneity of microglial activation in the innate immune response in the brain. J. Neuroimmune Pharmacol..

[19] Zhou X., Spittau B., Krieglstein K. (2012). TGFβ signalling plays an important role in IL4-induced alternative activation of microglia. J. Neuroinflamm..

[20] Zhou X., Zöller T., Krieglstein K., Spittau B. (2015). TGFβ1 inhibits IFNγ-mediated microglia activation and protects mDA neurons from IFNγ-driven neurotoxicity. J. Neurochem..

[21] Zlotnik A., Spittau B. (2014). GDNF fails to inhibit LPS-mediated activation of mouse microglia. J. Neuroimmunol..

[22] Keren-Shaul H., Spinrad A., Weiner A., Matcovitch-Natan O., Dvir-Szternfeld R., Ulland T.K., David E., Baruch K., Lara-Astaiso D., Toth B. (2017). A Unique Microglia Type Associated with Restricting Development of Alzheimer’s Disease. Cell.

[23] Holtman I.R., Raj D.D., Miller J.A., Schaafsma W., Yin Z., Brouwer N., Wes P.D., Möller T., Orre M., Kamphuis W. (2015). Induction of a common microglia gene expression signature by aging and neurodegenerative conditions: A co-expression meta-analysis. Acta Neuropathol. Commun..

[24] Butovsky O., Weiner H.L. (2018). Microglial signatures and their role in health and disease. Nat. Rev. Neurosci..

[25] Zöller T., Attaai A., Potru P.S., Ruß T., Spittau B. (2018). Aged Mouse Cortical Microglia Display an Activation Profile Suggesting Immunotolerogenic Functions. Int. J. Mol. Sci..

[26] Kamphuis W., Kooijman L., Schetters S., Orre M., Hol E.M. (2016). Transcriptional profiling of CD11c-positive microglia accumulating around amyloid plaques in a mouse model for Alzheimer’s disease. Biochim. Biophys. Acta.

[27] Krasemann S., Madore C., Cialic R., Baufeld C., Calcagno N., El Fatimy R., Beckers L., O’Loughlin E., Xu Y., Fanek Z. (2017). The TREM2-APOE Pathway Drives the Transcriptional Phenotype of Dysfunctional Microglia in Neurodegenerative Diseases. Immunity.

[28] Liu J., Wu Q., Shi J., Guo W., Jiang X., Zhou B., Ren C. (2020). LILRB4, from the immune system to the disease target. Am. J. Transl. Res..

[29] Bąbolewska E., Brzezińska-Błaszczyk E. (2012). Mast cell inhibitory receptors. Postepy Hig. Med. Dosw..

[30] Park M., Liu R.W., An H., Geczy C.L., Thomas P.S., Tedla N. (2017). A dual positive and negative regulation of monocyte activation by leukocyte Ig-like receptor B4 depends on the position of the tyrosine residues in its ITIMs. Innate Immun..

[31] Zhou J.S., Friend D.S., Lee D.M., Li L., Austen K.F., Katz H.R. (2005). gp49B1 deficiency is associated with increases in cytokine and chemokine production and severity of proliferative synovitis induced by anti-type II collagen mAb. Eur. J. Immunol..

[32] Qiu T., Zhou J., Wang T., Chen Z., Ma X., Zhang L., Zou J. (2019). Leukocyte immunoglobulin-like receptor B4 deficiency exacerbates acute lung injury via NF-κB signaling in bone marrow-derived macrophages. Biosci. Rep..

[33] Garcia J.A., Cardona S.M., Cardona A.E. (2014). Isolation and analysis of mouse microglial cells. Curr. Protoc. Immunol..

[34] Koerten H.K., Ploem J.S., Daems W.T. (1980). Ingestion of latex beads by filopodia of adherent mouse peritoneal macrophages. A scanning electron microscopical and reflection contrast microscopical study. Exp. Cell Res..

[35] Wagner M., Koester H., Deffge C., Weinert S., Lauf J., Francke A., Lee J., Braun-Dullaeus R.C., Herold J. (2014). Isolation and intravenous injection of murine bone marrow derived monocytes. J. Vis. Exp..

[36] Chen X., Liu Z., Cao B.-B., Qiu Y.-H., Peng Y.-P. (2017). TGF-β1 Neuroprotection via Inhibition of Microglial Activation in a Rat Model of Parkinson’s Disease. J. Neuroimmune Pharmacol..

[37] Taylor R.A., Chang C.-F., Goods B.A., Hammond M.D., Mac Grory B., Ai Y., Steinschneider A.F., Renfroe S.C., Askenase M.H., McCullough L.D. (2017). TGF-β1 modulates microglial phenotype and promotes recovery after intracerebral hemorrhage. J. Clin. Investig..

[38] Jiang Z., Qin J.-J., Zhang Y., Cheng W.-L., Ji Y.-X., Gong F.-H., Zhu X.-Y., Zhang Y., She Z.-G., Huang Z. (2017). LILRB4 deficiency aggravates the development of atherosclerosis and plaque instability by increasing the macrophage inflammatory response via NF-κB signaling. Clin. Sci..

[39] Dresselhaus E.C., Meffert M.K. (2019). Cellular Specificity of NF-κB Function in the Nervous System. Front. Immunol..

[40] Horvat A., Schwaiger F., Hager G., Brocker F., Streif R., Knyazev P., Ullrich A., Kreutzberg G.W. (2001). A novel role for protein tyrosine phosphatase shp1 in controlling glial activation in the normal and injured nervous system. J. Neurosci..

[41] Zhao J., Brooks D.M., Lurie D.I. (2006). Lipopolysaccharide-activated SHP-1-deficient motheaten microglia release increased nitric oxide, TNF-alpha, and IL-1beta. Glia.

[42] Park M., Raftery M.J., Thomas P.S., Geczy C.L., Bryant K., Tedla N. (2016). Leukocyte immunoglobulin-like receptor B4 regulates key signalling molecules involved in FcγRI-mediated clathrin-dependent endocytosis and phagocytosis. Sci. Rep..

[43] Salih D.A., Bayram S., Guelfi S., Reynolds R.H., Shoai M., Ryten M., Brenton J.W., Zhang D., Matarin M., Botia J.A. (2019). Genetic variability in response to amyloid beta deposition influences Alzheimer’s disease risk. Brain Commun..

[44] Kaiser T., Feng G. (2019). Tmem119-EGFP and Tmem119-CreERT2 Transgenic Mice for Labeling and Manipulating Microglia. eNeuro.

[45] Masuda T., Amann L., Sankowski R., Staszewski O., Lenz M., d´Errico P., Snaidero N., Costa Jordão M.J., Böttcher C., Kierdorf K. (2020). Novel Hexb-based tools for studying microglia in the CNS. Nat. Immunol..

